# Cellulose Biofilms, New Biotemplates in the Synthesis of Cuprate Superconductors

**DOI:** 10.1021/acsomega.6c00196

**Published:** 2026-05-28

**Authors:** Jan Maurycy Uszko, Safiyyah Adedoyin Abibu, Stephen J. Eichhorn, Avinash J. Patil, Simon R. Hall

**Affiliations:** † The Bristol Composites Institute (BCI), School of Civil, Aerospace and Design Engineering University Walk, 1980 University of Bristol, Bristol BS8 1TR, U.K.; ‡ School of Chemistry, 1980 University of Bristol, Cantock’s Cl, Bristol BS8 1TS, U.K.

## Abstract

Bacterial cellulose (BC), obtained from fermented food byproducts (Symbiotic Culture of Bacteria and Yeast, and Nata de Coco), was successfully used as a template for the synthesis of a YBa_2_Cu_3_O_6+δ_ (YBCO) superconductor. As previous studies have shown, a dry template is needed to ensure the maximum uptake of the precursor solution. BC used is obtained in a wet state; it must be dried before use as a template. A variety of template drying techniques were investigated to assess the efficacy. This included air, oven, freeze, and solvent exchange drying. Among these, freeze-drying proved to be the most effective method as it best preserved the porous internal structure of the template. The addition of ethylenediaminetetraacetic acid (EDTA), a polychelating acid, also had a beneficial effect on the synthesis, improving both phase purity and the contribution of the superconducting phase. Waste-derived BC was shown to be a suitable substrate for the sol–gel synthesis of cuprate superconductors, providing an alternative to the ionic-liquid/nanocellulose-based approach used previously.

## Graphical abstract

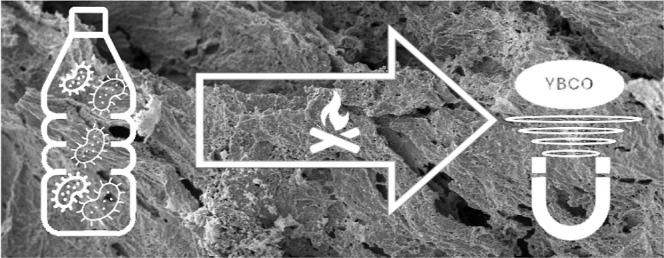

## Introduction

The sol–gel method for the synthesis of complex metal oxide superconductors provides significant improvements over conventional solid-state syntheses. The use of polymers and aqueous precursors simplifies the process, reducing both time and complexity.^1^ These improvements arise mainly from addressing one of the biggest problems of solid-state synthesis, the lack of homogeneity of the starting material. The use of the sol–gel method for the synthesis of superconductors is constantly evolving from using traditional biopolymers, to commercially available synthetic sponges and recently to natural templates.^2–5^ These developments aim to improve sustainability, enhance morphological control, and further reduce synthesis complexity.

Cellulose, the most abundant organic polymer on earth, has been used for millennia. Since its discovery in the 1950s, nanocellulose has gained more and more popularity.^6^ It is valued for its biodegradability, bioavailability, mechanical properties, and large surface area.^7^ The presence of hydrogen bonding allows cellulose to be used as a weakly coordinating, polyanionic, nonspecific chelating agent.^2^ However, the method, described by Green et al., requires highly processed crystalline nanocellulose and the use of ionic liquids, an expensive and potentially hazardous solvent.^2,8^ To address these limitations, this work modifies two key aspects of the method: replacing the high-purity commercial crystalline nanocellulose with a more sustainable and readily available biotemplate and eliminating the need for ionic liquids to develop a safer, more cost-effective, and environmentally friendly synthesis route.

A promising alternative is bacterial cellulose (BC). Unlike plant-derived cellulose, BC does not hold a structural function. No need for lignin and hemicellulose resulted in a very pure form of cellulose that requires less preprocessing.^9^ Its properties, including biocompatibility, bioavailability, mechanical strength, water retention, and high porosity, make it attractive in various applications.^10–12^ BC is grown by microorganisms at the air–liquid interface, notably the Komagataeibacter xylinus, forming into a three-dimensional, nanofibrillar network.^10,13^ In this work, BC was sourced from two food-related products: Symbiotic Culture of Bacteria and Yeast (SCOBY) and Nata de Coco (NDC). SCOBY is produced during the fermentation of tea in the process of making kombucha.^14^ The side product of the reaction includes various organic acids, which might have beneficial chelating properties.^15^ NDC is a sweet-tasting dessert consumed in the Philippines and is produced commercially via the fermentation of coconut water and later preservation in sugar syrup.^16^ These materials originate from culinary waste, either as a byproduct in the case of SCOBY or the ingredient itself for NDC, so that the use of kitchen waste provides a unique opportunity to upcycle food BC into high-value materials. Given the potential benefits of using BC and the ever-increasing need for superconductors, this work will focus on investigating whether BC originating from food waste can be used as a biotemplate for cuprate-based superconductors.

Fibrillated forms of cellulose, particularly “nanocelluloses” are known to undergo a form of irreversible aggregation upon drying called “hornification”, a process which is still trying to be better understood.^17^ The use of different drying methods is known to result in varying degrees of aggregation, and solvent exchange has been shown to have some positive effects in this respect, whereby reversible aggregation can be achieved to some extent.^18^ There have been some attempts to produce rehydratable forms of bacterial cellulose, including recently the use of charged polysaccharides such as sodium alginate.^19^ The different methods for drying nanocelluloses have also been recently reviewed, summarizing the various advantages and disadvantages.^20^ Previous work on using biotemplates in sol–gel synthesis of cuprate superconductors utilized dry templates, which were infused with the precursor solution.^5^ BC being grown in an aqueous environment will therefore require preinfusion drying treatments to optimize the yield and phase purity of the end product.

## Results and Discussion

To assess the influence of drying methods on biotemplate integrity and YBa_2_Cu_3_O_6+δ_ (YBCO) formation, the BC templates were subjected to five different treatments. Treated samples were labeled as nondried, air-dried, oven-dried, freeze-dried, and solvent-exchanged.^21^ Each technique induced distinct physical, structural, and chemical changes that were reflected in shrinkage, swelling capacity, and ultimately the yield and phase purity of the final YBCO product.

Shrinkage was calculated using eq 1 and is shown in Figure 1. It serves as an indirect indicator of structural collapse, which can affect porosity and accessibility for metal ion infiltration. SCOBY exhibited high shrinkage in the oven-, air-, and freeze-dried conditions, while solvent exchange showed moderate shrinkage, consistent with it being a soft drying technique. NDC samples exhibit a similar behavior but with a lower overall shrinkage for all methods. These differences likely arise from the internal structure of each template, with SCOBY’s dense fibrous network being more prone to collapse, while the NDC matrix retains more of its original volume.

1 $$shrinkage=\left(1-\frac{dryweight}{wetweight}\right)\cdot 100$$

**Figure 1:**
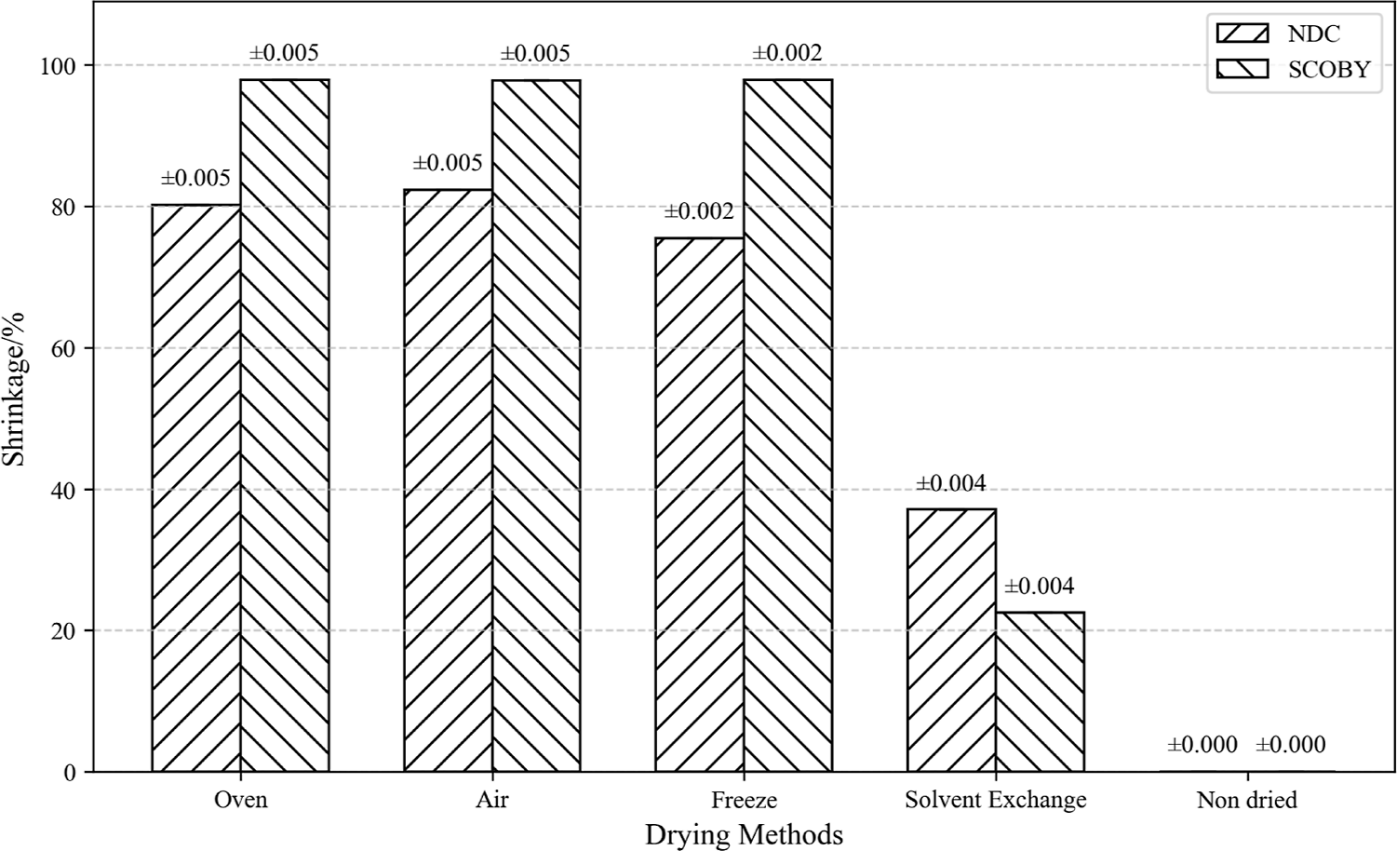
Shrinkage of SCOBY and NDC subjected to different drying methods.

The swelling degree described in eq 2 shown in Figure 2 reflects how effectively a dried scaffold can reabsorb liquid, which is essential for precursor dispersion. Freeze-dried SCOBY achieved a remarkable swelling far exceeding that of air- and oven-dried counterparts. This highlights freeze-drying as the most effective method for retaining rehydration capacity and porosity for this material. For NDC, the highest swelling ratio was observed in air-dried samples, followed by solvent-exchanged, oven-dried, and freeze-dried. Interestingly, the freeze-dried NDC showed a much lower swelling ratio compared to its SCOBY equivalent, suggesting that NDC’s gel-like matrix may not retain porous structure as efficiently.^22^

2 $$swelling=\left(\frac{infusedweight-dryweight}{dryweight}\right)\cdot 100$$

Furthermore, both nondried samples of NDC and SCOBY produced negative degrees of swelling, which suggests a contraction likely caused by chelation around the metal precursor ions as they displace water molecules within the biotemplate.^23^

**Figure 2:**
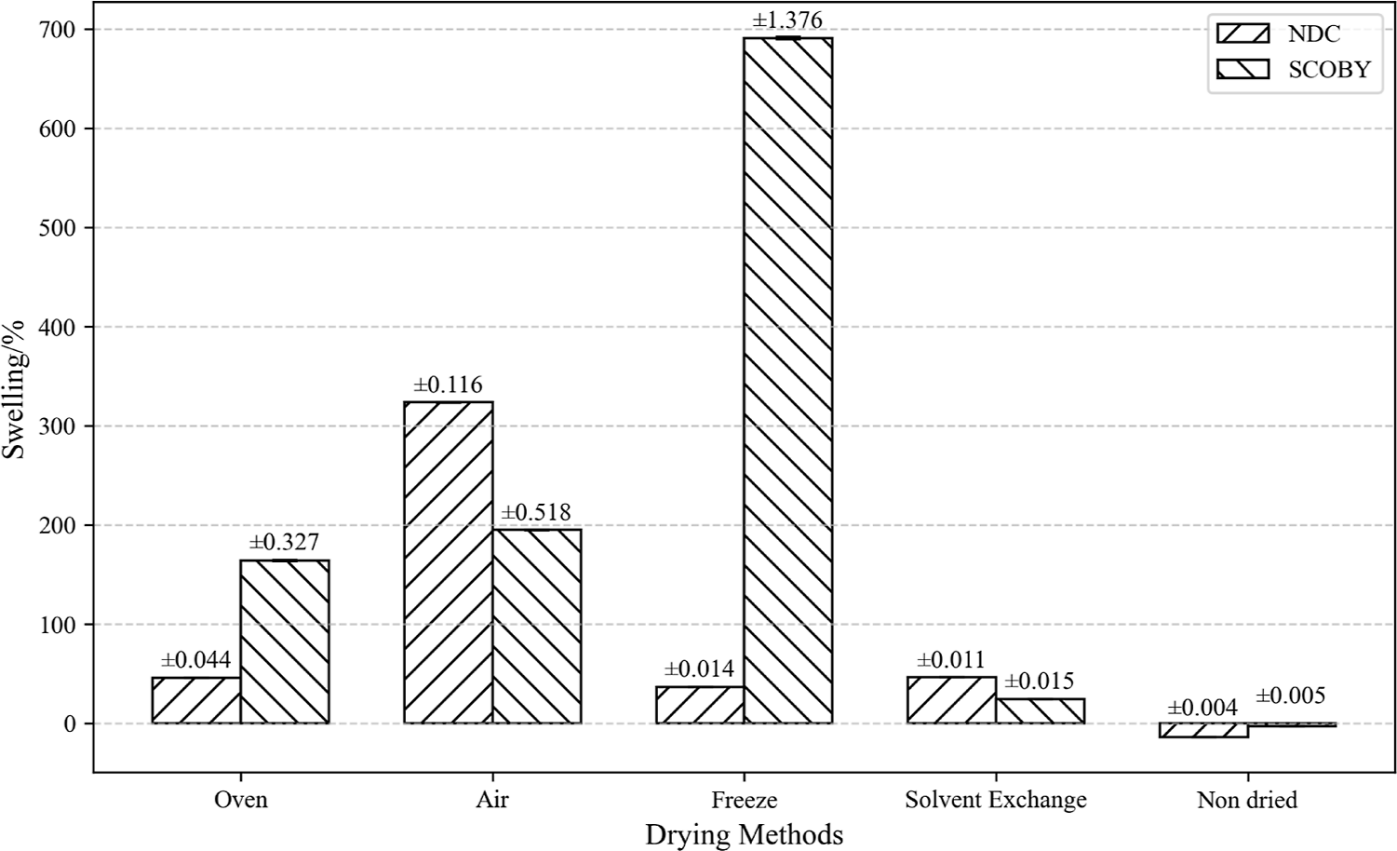
Swelling of SCOBY and NDC subjected to different drying methods.

SEM micrographs in Figure 3 show the structure of YBCO templated with SCOBY and NDC. The presence of pores has a beneficial effect in the synthesis of superconductors as increased surface area allows for better oxygenation of the precursor during calcination.^24^ In the final superconductor, a controlled porous architecture can also enhance cryogen penetration and heat dissipation, thereby reducing the likelihood of hot spots.^4,5^ Nondried samples in Figures 3a and 3g show a well-preserved, three-dimensional porous network composed of a multilayered structure. For NDC, pores are less uniform and more flake-like. In contrast, air-dried samples in Figure 3b,h display moderate structural shrinkage with a partially collapsed fibrillar network with some structural features retained. Although the overall porosity is still evident, the pores appear more compressed and less defined than those in the nondried counterpart. Oven-dried examples, Figure 3d,j, presents a dense and significantly compacted microstructure, characterized by flattened surfaces and diminished pore visibility. This indicates a complete collapse of the cellulose framework during thermal dehydration. The NDC sample shows the presence of a flake-like microstructure similar to the one present in the nondried sample. The freeze-dried sample, templated with SCOBY in Figure 3c exhibited the most open and interconnected architecture among the drying methods. The structure maintained large and well-defined voids with both smooth and flaky textures. On the other hand, NDC in Figure 3i exhibits a fragmented, flaky sheet-like structure. While traditional circular pores are largely absent, the sample reveals numerous elongated linear features. This is consistent with the difference in swelling described previously. The samples dried by solvent exchange, Figure 3e,k, reveal a wrinkled and uneven surface with a mixture of variously sized pores. The effect of using EDTA shown in Figure 3f,l is similar in both cases. The sample displayed a heterogeneous surface composed of flat, compacted regions interspersed with highly fibrous and porous zones. The chelating effect of EDTA likely modified the coordination environment during synthesis, leading to a mixture of dense and porous morphologies. Pores measured were relatively small compared to those of the nondried.

**Figure 3:**
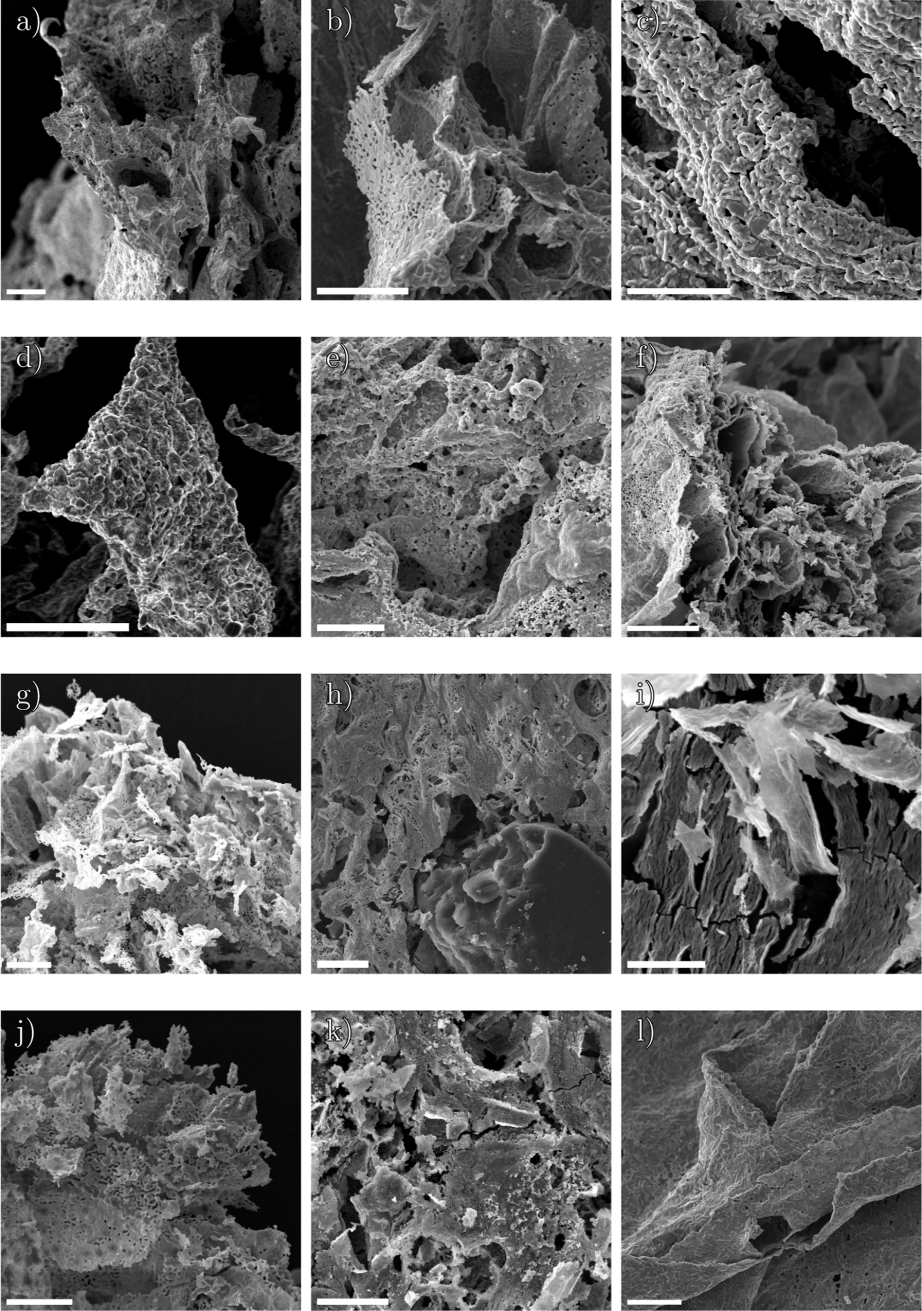
SEM micrographs of YBCO synthesized using templates subjected to various drying and chemical treatment methods. The images highlight structural variations arising from (a) nondried, (b) air-dried, (c) freeze-dried, (d) oven-dried, (e) solvent exchange, and (f) EDTA-modified templated with SCOBY and (g) nondried, (h) air-dried, (i) freeze-dried, (j) oven-dried, (k) solvent exchange, and (l) EDTA-modified templated with NDC. All scale bars are 20 μm.

The energy-dispersive X-ray (EDX) analysis confirmed the presence of yttrium, barium, and copper in all samples, as well as trace amounts of calcium and magnesium. The trace elements most likely are present in non-DI water used to brew kombucha; Bristol tap water is known for its high hardness level.^25^ It is postulated that hard water was also the source of calcium and magnesium in NDC. After confirming the elemental composition, powder X-ray diffraction patterns (PXRD) were collected (Figures 4 and 5) to confirm the composition of the prepared materials. The effect of adding EDTA can be observed in Figure 6. The phase purity of each sample is illustrated in Table 1; collection codes for the phases used in Rietveld refinement can be found in Table 2.

**Figure 4:**
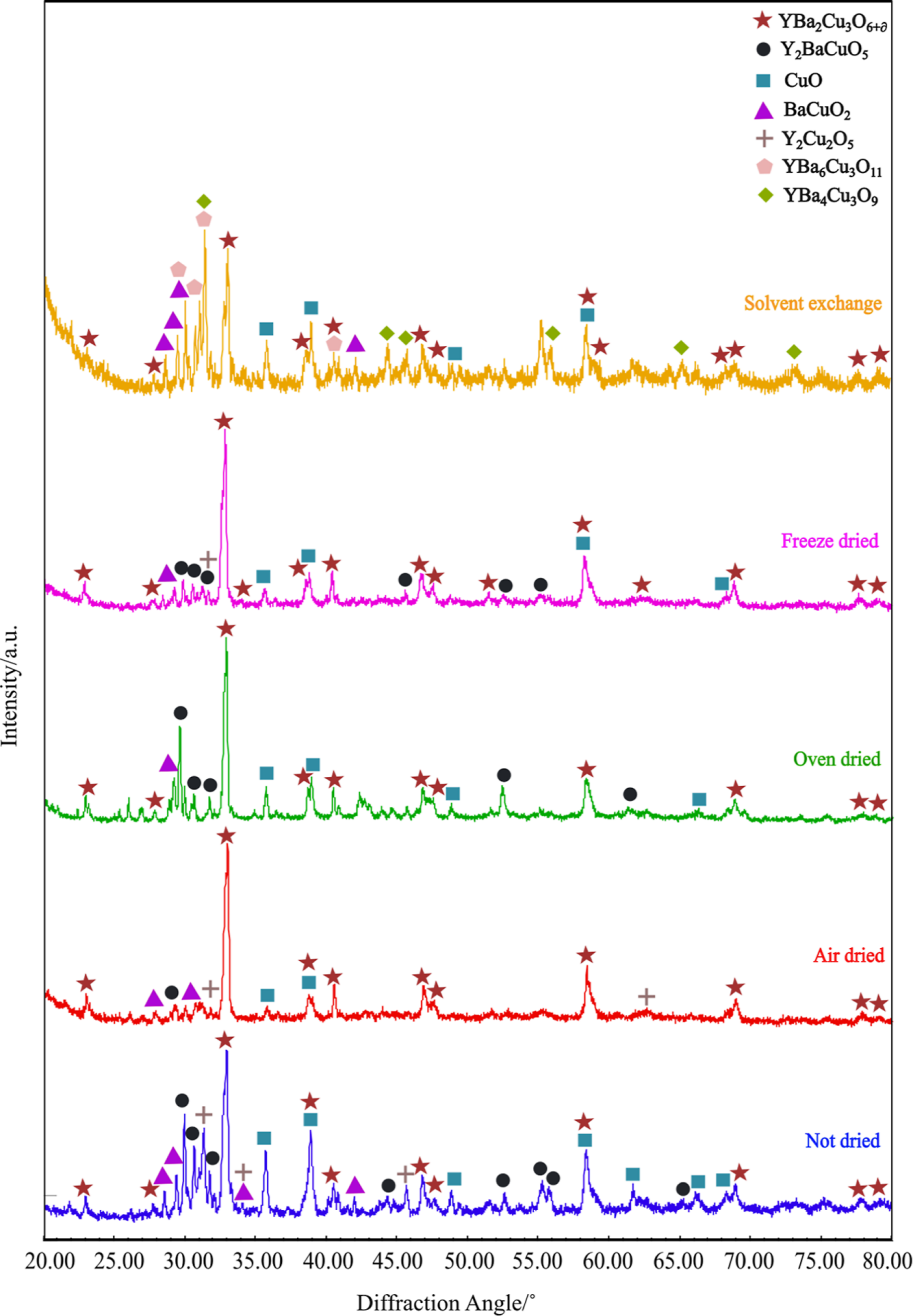
PXRD patterns of YBCO synthesized using SCOBY subjected to different drying methods.

**Figure 5:**
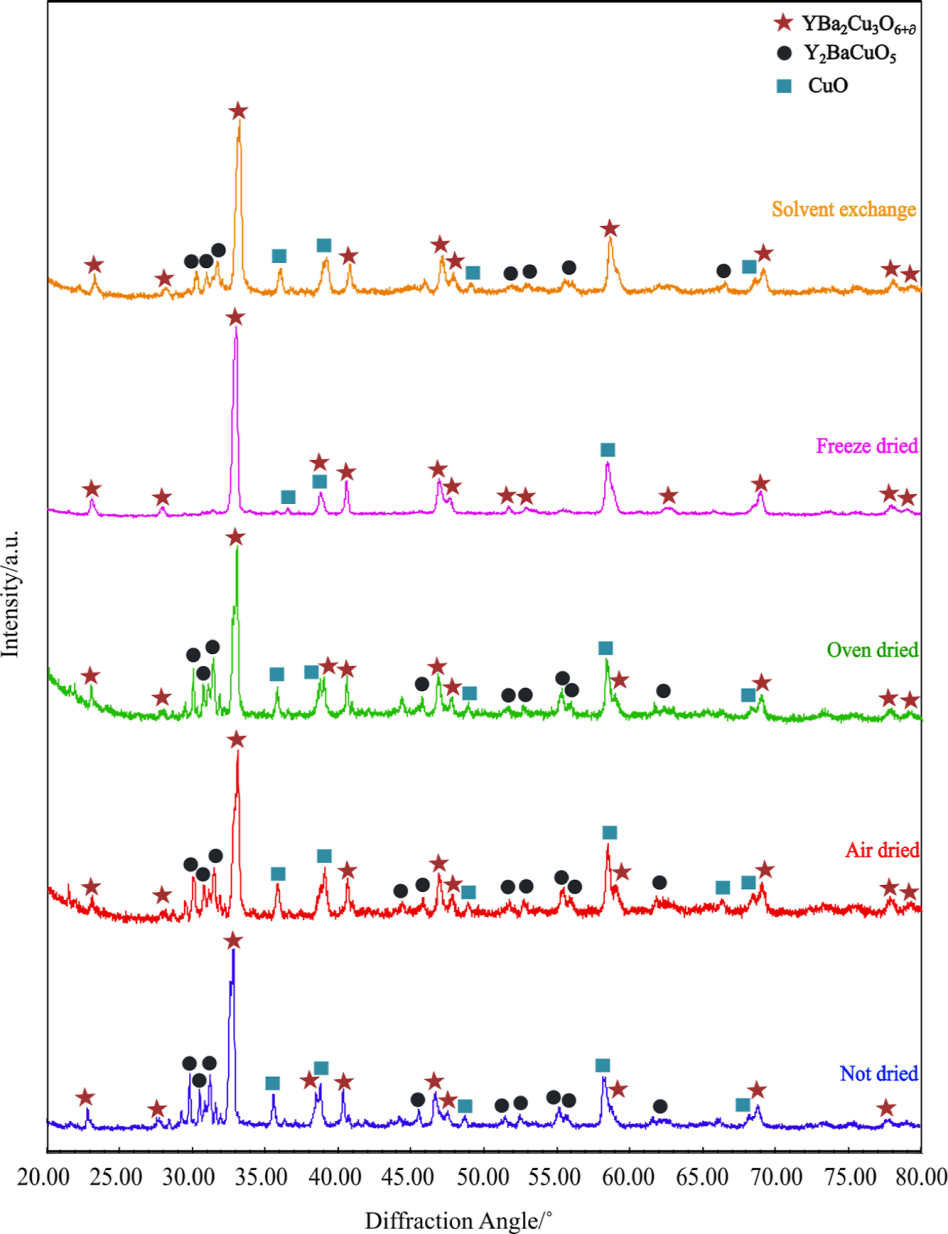
PXRD patterns of YBCO synthesized using NDC subjected to different drying methods.

**Figure 6:**
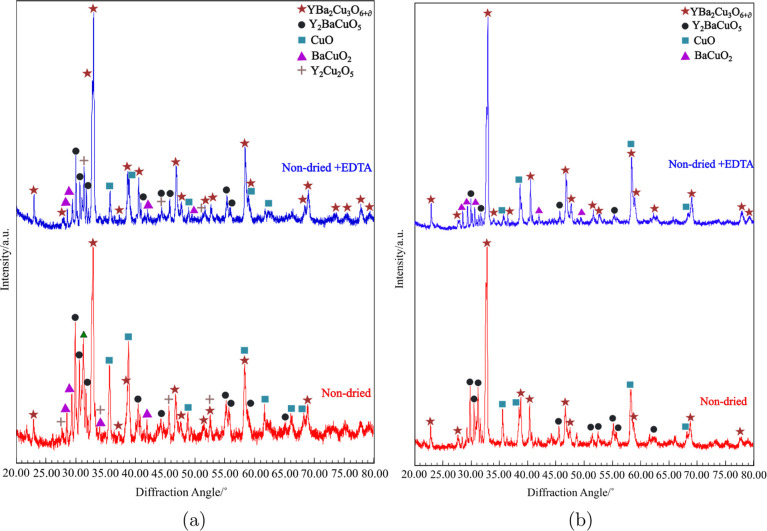
Comparison of PXRD patterns of YBCO synthesized with and without EDTA, samples were templated with (a) SCOBY, (b) NDC.

**Table 1:** YBCO Contribution in the Prepared Samples and χ^2^ Values for the Rietveld Refinement

SCOBY		
pretreatment	YBa_2_Cu_3_O_6+δ_ contribution/%	χ^2^
nondried	34.5	4.14
air-dried	70.3	2.65
oven-dried	69.5	4.88
freeze-dried	54.0	1.86
solvent exchange	28.7	2.72
EDTA	56.8	2.70
NDC		
nondried	56.2	5.54
air-dried	52.5	3.02
oven-dried	45.4	3.71
freeze-dried	90.8	1.83
solvent exchange	59.1	3.26
EDTA	70.6	2.64

Among all SCOBY samples, the freeze-dried SCOBY exhibited the lowest χ^2^ value (1.86), indicating the best fit to a recognized crystal structure. This suggests both high phase selectivity and good crystallinity. The minimal structural collapse during freeze-drying likely helped preserve the porous architecture of the SCOBY scaffold, enhancing precursor infiltration and uniform ion distribution. While the sol–gel method inherently provides molecular-level precursor homogeneity, the effectiveness of this chemistry within the scaffold depends strongly on preserved pore connectivity, which is maximized in the freeze-dried templates. However, secondary phases including CuO, Y_2_BaCuO_5_, Y_2_Cu_2_O_5_, and BaCuO_2_ were still present, suggesting incomplete conversion. The air-dried SCOBY also performed well, with a combined YBCO phase and a moderate χ^2^ value. This indicates a reasonable structural match, though the presence of multiple YBCO phases and several secondary phases, most notably Y_2_Cu_2_O_5_ and Y_2_BaCuO_5_, suggests phase heterogeneity. This may reflect partial preservation of the SCOBY network but less control over uniform metal ion distribution compared to freeze-drying. In contrast, oven-dried SCOBY yielded a similar YBCO contribution but exhibited a much higher χ^2^ value, a worse structural match. The result supports the interpretation that thermal drying induced significant collapse of the biotemplate’s internal structure, which impeded homogeneous phase formation. The elevated amounts of Y_2_BaCuO_5_ and CuO further support incomplete reaction or nonuniform metal incorporation. The SCOBY dried via solvent exchange exhibited the poorest superconducting phase development and a χ^2^ value of 2.72. Despite a moderate refinement fit, the sample contained numerous complex secondary phases including YBa_6_Cu_3_O_11_ and YBa_4_Cu_3_O_9_ as well as a large proportion of CuO. These results may be due to the effects from the residual organic solvents used that may have a negative impact on the YBCO synthesis. The nondried SCOBY templated sample demonstrated a relatively low yield of the YBCO phase with a χ^2^ value of 4.14, indicating a moderate-quality fit between observed and calculated patterns.

Among the NDC-templated samples, the freeze-dried sample yielded the most promising result, achieving a high YBCO content and the lowest χ^2^ value (1.83). The low value suggests excellent crystallinity and high phase purity. The minimal structural collapse associated with freeze-drying likely preserved the 3D network of the NDC biotemplate, promoting efficient precursor infiltration and homogeneous ion distribution.^32^ This, in turn, seems to have favored the formation of the desired YBCO phase while minimizing the generation of the impurity phases such as CuO. In contrast, air-dried and oven-dried NDC samples exhibited significantly lower YBCO yields, with higher levels of the impurity phases of CuO and Y_2_BaCuO_5_. Air- and oven-drying likely induced further structural collapse, restricting pore size and ion distribution.^32^ Both samples also recorded relatively higher χ^2^ values, indicating slightly poorer fits. The nondried NDC sample produced a moderate YBCO content with a relatively high χ^2^ value of 5.54, the largest among all NDC conditions. The secondary phase content remained substantial, suggesting incomplete conversion of precursor materials. This may stem from retained organic residues such as sugars and microbial byproducts from the fermentation process that interfere with the coordination chemistry and chelation of metal cations during soaking, ultimately disrupting phase formation and crystallization during calcination. The NDC sample dried via solvent-exchange produced a moderate YBCO content along with CuO and Y_2_BaCuO_5_ phases. Despite a χ^2^ value indicating a moderately successful refinement, the increased impurity content suggests that solvent effects from the residual drying solvents may have a negative impact on the YBCO synthesis.

The EDTA-treated samples displayed increased YBCO content and improved χ^2^ values, indicating enhanced phase purity. EDTA likely promoted more controlled metal chelation, leading to improved precursor homogeneity.

Samples with high purity, templated with SCOBY and NDC, were selected for further analysis. Superconducting quantum interference device (SQUID) magnetometry measurements in Figure 7 were used to analyze the magnetic behavior of YBCO prepared from freeze-dried NDC and SCOBY templates. The results confirmed superconducting behavior, with transition temperatures (*T*_c_) consistent with YBCO. The slight variation in *T*_c_ between templates likely arises from incomplete optimization of oxygenation.^33^

**Figure 7:**
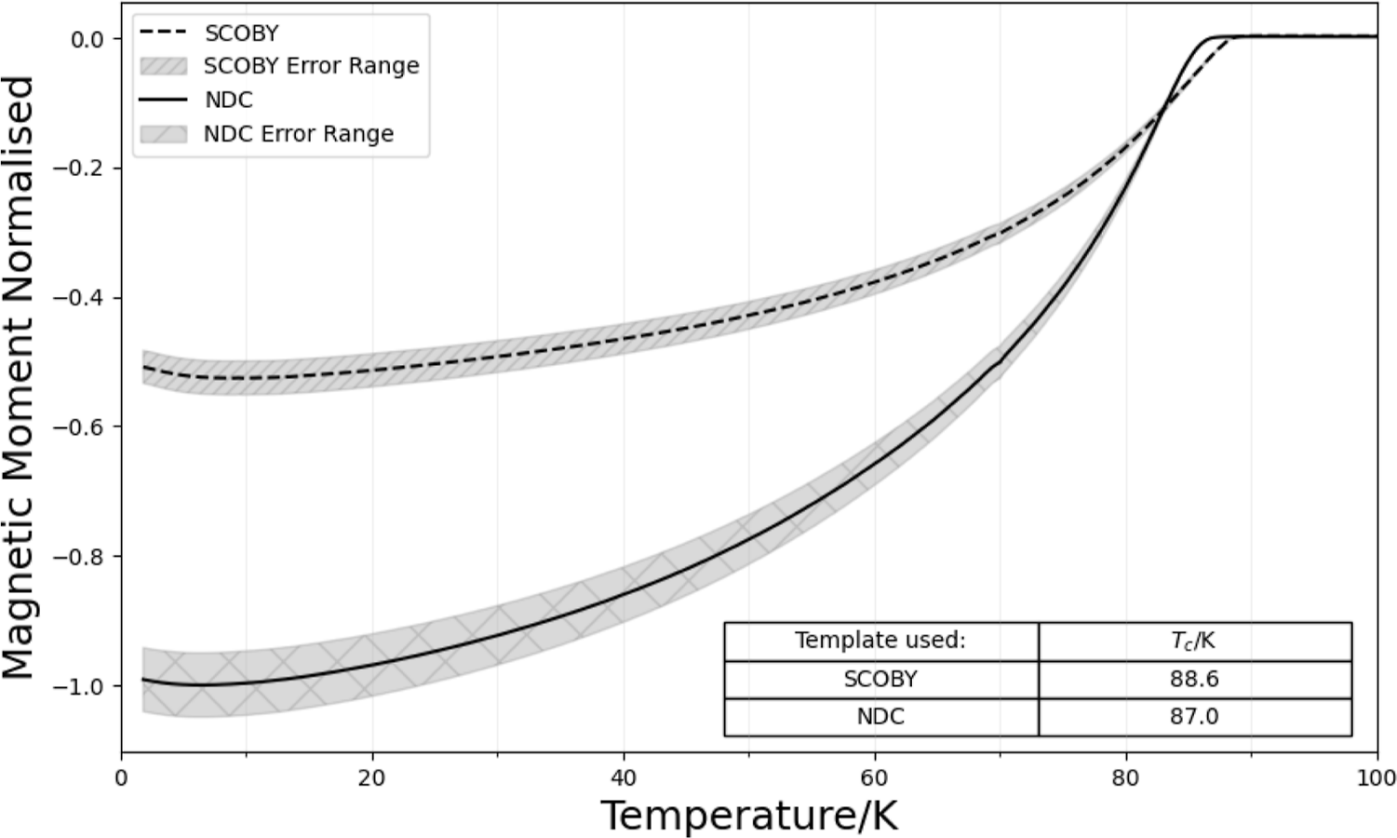
SQUID magnetometry measurements of YBCO synthesized using freeze-dried NDC and SCOBY templates, showing the superconducting transition temperature (*T*_c_).

## Conclusions

BC derived from food waste has been successfully used as a sol–gel template to produce cuprate-based superconductors. The use of BC retains the benefits of the sol–gel synthesis while improving on previous attempts at templating with cellulose by substituting ionic liquids with water and highly processed nanocellulose with waste-derived BC. This supports the goal of democratizing materials chemistry by lowering the initial cost requirements for the synthesis.

This work also highlights some difficulties arising from the upcycling of waste BC into high-value superconductors. The use of tap water during biotemplate preparation, chosen to reflect realistic low-cost and food-derived processing conditions, introduced inorganic contamination that persisted in the final product.

The addition of EDTA was found to enhance the phase purity of the final materials. Among the drying methods tested, freeze-drying proved to be the most effective in preserving an open structure, enabling the effective diffusion of ions and resulting in the highest phase purity.

SQUID magnetometry measurements confirmed the superconducting behavior of the synthesized materials, with transition temperatures consistent with YBCO (see Figure 7).

## Experimental Section

SEM analysis was performed on a JEOL JSM-IT300 system. TEM analysis was performed on a JEOL JEM-2100 system. Powder X-ray diffraction (PXRD) patterns were acquired on a Bruker D8 Advance powder X-ray diffractometer equipped with a PSD LynxEye detector and utilizing Cu–Kα radiation (λ = 1.5418 Å). Multiphase Rietveld refinement was performed in Profex 5.3.0.1.^34^ Magnetometry analysis was performed using Quantum Design MPMS3. The Inorganic Crystal Structure Database (ICSD) reference numbers for files used in the Rietveld refinement can be found in Table 2.

**Table 2:** Inorganic Crystal Structure Database (ICSD) Collection Codes for Phases Used in Rietveld Refinement

phase	ICSD collection code
CuO	257091 ^26^
Y_2_BaCuO_5_	108831 ^27^
YBa_2_Cu_3_O_6.89_	83208 ^28^
YBa_4_Cu_3_O_9_	65549 ^29^
YBa_6_Cu_3_O_11_	65550 ^29^
BaCuO_2_	1049 ^30^
Y_2_Cu_2_O_5_	202877 ^31^

### Template Preparation

To 1 L of boiling water were added 15 g of black tea and 225 g of sugar. The solution was stirred until the sugar had fully dissolved, and the tea had brewed enough to give a dark amber color. The solution was filtered into a 5 L glass jar, and 3 L of water was added. The solution was allowed to cool to room temperature before adding 0.5 L of the kombucha starter and SCOBY pellet. The pH of the solution was controlled and adjusted with lemon juice at around 4. The glass jar was covered with a breathable cotton cloth. Fermentation progress was monitored by tasting the liquid and observing the SCOBY growth. After 10 days, SCOBY and 0.5 L of Kombucha were transferred to a smaller jar and transported to the lab for templating. NDC was washed with DI water to remove the sugar residues.

### YBCO Precursor

A YBCO precursor solution was prepared as described by Uszko et al.^5^ In short, metal salts described in Table 3 were dissolved in DI water in selected experiments. 0.0068 M EDTA was included as a chelating agent.

**Table 3:** Materials Used and the Composition of Precursor Solution for 10 mL

templates		
material	amount	supplier
Kombucha Starter	500 mL	Kombuchaorganic Store
Nata de Coco	340 g	Buenas
precursor solution		
material	amount/g	supplier
Y(NO_3_)_3_ · 6 H_2_O	0.192	Sigma-Aldrich
Ba(NO_3_)_2_	0.261	Sigma-Aldrich
Cu(NO_3_)_2_·2.5 H_2_O	0.349	Sigma-Aldrich

### Template Treatment

Both SCOBY and NDC were cut into uniform cubes (2 cm) with a scalpel and subjected to a variety of drying and pretreatment techniques described in Table 4. After pretreatment, samples were submerged in precursor solution and infused for 24 h.

**Table 4:** Pretreatment Techniques Used on the Template before Infusion with the YBCO Precursor

technique	description
oven-drying	sample placed in a drying oven at 100 °C for 9 h
air-drying	sample left uncovered under ambient conditions (21 °C) for 7 days
freeze-drying	sample frozen using liquid nitrogen and dried under vacuum
solvent-exchange	sample sequentially immersed in ethanol, acetone, and heptane (5 min per solvent)

### Calcination

Infused templates were calcined at 920 °C, with a ramp rate of 5 °C min^–1^ and a dwell time of 4 h in uncovered alumina crucibles in air.
